# Supplementary material for: Herbicide Bioassay Using a Multi-Well Plate and Plant Spectral Image Analysis
Source: Sensors (Basel). 2024 Jan 31;24(3):919. doi: 10.3390/s24030919 (PMC10856836; doi:10.3390/s24030919)
Supplement: Supplementary file 1 [file sensors-24-00919-s001.zip › sensors-2833551-supplementary.pdf]

Table S1. The proportion of variance that the first and second principal component (PC) explained (% of total variance).

Time (HAT)	Proportion of Variance (% of total variance)		
	**PC1**	**PC2**	**Sum of explained variance**
**3**	62.7	19.7	82.5
**6**	77.5	16.6	94.1
**24**	85.6	10.0	95.6
**48**	75.8	18.2	94.0
**72**	89.9	7.0	96.9
**120**	84.4	9.9	94.3
**Pooling**	69.6	14.7	84.3

Table S2. The proportion of variance that the first, second, and third principal component (PC) explained (% of total variance).

Time (HAT)	Proportion of variance (% of total variance)			
	**PC1**	**PC2**	**PC3**	**Sum of explained variance**
**3**	62.7	19.7	11.3	93.8
**6**	77.5	16.6	5.3	99.4
**24**	85.6	10.0	3.0	98.5
**48**	75.8	18.2	3.6	97.6
**72**	89.9	7.0	2.4	99.2
**120**	84.4	9.9	4.6	98.8
**Pooling**	69.6	14.9	4.1	88.4

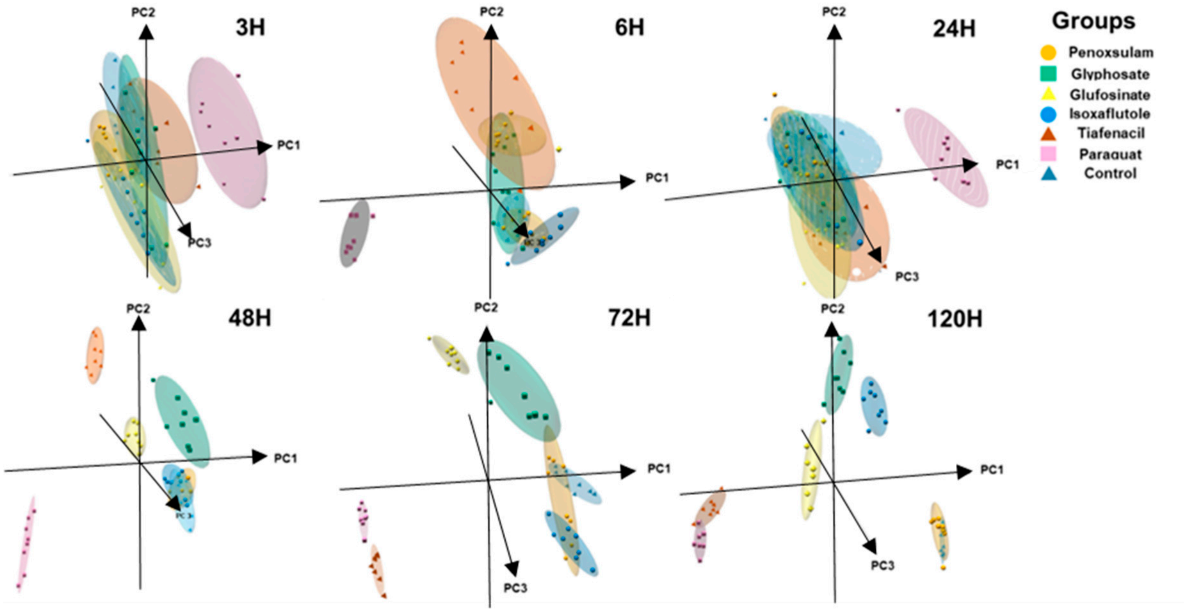

Figure S1. PCA results of six spectral parameters of crabgrass at 3 h, 6 h, 24 h, 48 h, 72 h, and 120 h after treatment of herbicides with different modes of action. These images represent PCA results using the PC1, PC2, and PC3 axes, and each symbol represents the replication.

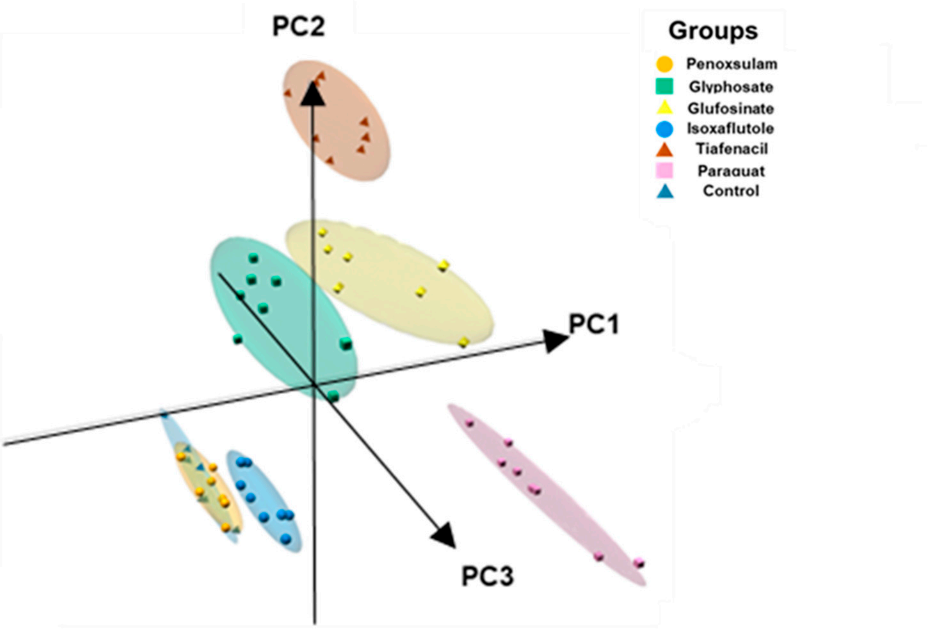

Figure S2. PCA results of six spectral parameters of crabgrass by aggregating all of the data including 3 h, 6 h, 24 h, 48 h, 72 h, and 120 h after treatment of herbicides with different modes of action. These images represent PCA results using the PC1, PC2, and PC3 axes, and each symbol represents the replication.
